# Some American Hospitals

**Published:** 1892-01-23

**Authors:** 


### Some American Hospitals.

By Our Special Commissioner.

### IV.—DENVER.

This city is situated in the heart of a great mining district, and there has been an enormous development during the last ten years. At the present time the population numbers upwards of 100,000. It contains several hospitals, but only three of them are of much importance. These are the Union Pacific Hospital, St. Luke's Hospital, and the County Hospital.

St. Luke's Hospital is managed by the Presbyterian Church, the County Hospital being supported by the town authorities. Both these institutions have outgrown the accommodation originally provided, and at the date of our visit, new hospital buildings, on a new site, were being erected for the accommodation of the patients they contained, and we do not, therefore, think it fair to criticise or report on two old establishments which may be regarded as obsolete.

The Union Pacific Railway Hospital is certainly the best we have seen in the Western continent since we left Canada. It is a hospital on the pavilion principle, designed on the old H plan. Visitors are at once struck by the cleanliness and efficiency of every portion of this institution. It contains altogether 84 beds, which are retained for the exclusive use of the employés of the Union Pacific Railway. It is under the charge of Dr. Chambers and Dr. Taylor. And the fact that two resident physicians are employed testifies to the liberality and wisdom of the directors of the Union Pacific Railway, seeing that the hospital is situated a long way from the centre of the city, and that unless two resident medical officers were maintained, there would be no opportunity for them to get away for necessary exercise and recreation. As may be supposed, a great many accidents (many of them of a most serious character) are constantly being admitted, not only from the railway proper, but from the railway works, which are situated in the immediate neighbourhood of the hospital. The water of Denver and the district generally would seem to be very unwholesome, having regard to the fact that the medical wards contained an unusually large proportion of cases of typhoid fever. The dispensary is entirely undertaken by one of the Sisters of the Order of St. Francis, whom Dr. Chambers informed us was as efficient and capable as any pharmaceutist to be met with in America. The nursing and housekeeping are undertaken by Sisters of the Order of St. Francis, and the condition of the hospital in every department proved that the arrangements were good, and that the patients are more than usually well cared for. We congratulate the authorities of the Union Pacific Railway on the efficiency of the staff, and the excellence of all the arrangements.

It may not, perhaps, be uninteresting, in this connection, to state that, so far as can be ascertained, there seems to be no private system of training Sisters of the Holy Cross at Salt Lake City, or those of St. Francis at Denver in the duties of a skilled nurse. We think this is a great blot, and we hope that the authorities in question will take steps to secure that proper classes are formed, and that lectures are regularly given, together with practical instruction. If the Sisters, who are unusually intelligent, can under existing conditions produce the results we saw, it is fair to conclude that, with adequate training, they would make a very high type of hospital nurse.

A few words may be said of the system adopted by the Directors of the Union Pacific Railway, with regard to their employés, who number from 12,000 to 15,000. Each employé pays the company 40 cents per month (about 1s. 8d.), and is entitled to receive in return medical attendance, either at home or at the hospital, when he may be disabled by illness or accident. Should a man be taken to the hospital, he is kept there until he is cured and enabled to return to work. Instead of having a separate convalescent institution, the upper part of the hospital is given up entirely to the accommodation of convalescents.

In addition to the hospital at Denver, the Union Pacific Railway has established another at Ogden, containing 15 to 20 beds, and has further made arrangements with the authorities of the hospitals at Omaha and elsewhere for the reception of their employes. The railway pays to the hospital authorities for each case admitted from five to seven dollars per week. This system of provident medical relief seems to work admirably, and we shall hope to give a fuller account of it on another occasion. Meanwhile, it may be stated that there is a large medical staff employed, and that the Chief Surgeon is Mr. W. J. Galbraith, who is assisted by Divisional Surgeons, having under them a number of assistant medical officers. The Chief Surgeon gives his whole time to the service, and receives an income of from £1,000 to £1,200 per annum, we believe.

